# Wireless Concrete Strength Monitoring of Wind Turbine Foundations

**DOI:** 10.3390/s17122928

**Published:** 2017-12-16

**Authors:** Marcus Perry, Grzegorz Fusiek, Pawel Niewczas, Tim Rubert, Jack McAlorum

**Affiliations:** 1 Department of Civil & Environmental Engineering, University of Strathclyde, Glasgow G1 1XJ, UK; 2 Department of Electronic & Electrical Engineering, University of Strathclyde, Glasgow G1 1XQ, UK; g.fusiek@strath.ac.uk (G.F.); p.niewczas@strath.ac.uk (P.N.); tim.rubert@strath.ac.uk (T.R.); jack.mcalorum@strath.ac.uk (J.M.)

**Keywords:** concrete maturity, wireless sensing, neural networks, structural health monitoring, foundation design

## Abstract

Wind turbine foundations are typically cast in place, leaving the concrete to mature under environmental conditions that vary in time and space. As a result, there is uncertainty around the concrete’s initial performance, and this can encourage both costly over-design and inaccurate prognoses of structural health. Here, we demonstrate the field application of a dense, wireless thermocouple network to monitor the strength development of an onshore, reinforced-concrete wind turbine foundation. Up-to-date methods in fly ash concrete strength and maturity modelling are used to estimate the distribution and evolution of foundation strength over 29 days of curing. Strength estimates are verified by core samples, extracted from the foundation base. In addition, an artificial neural network, trained using temperature data, is exploited to demonstrate that distributed concrete strengths can be estimated for foundations using only sparse thermocouple data. Our techniques provide a practical alternative to computational models, and could assist site operators in making more informed decisions about foundation design, construction, operation and maintenance.

## 1. Introduction

Wind turbines currently supply 10% of Europe’s electricity [1], and as this proportion is only expected to increase [2,3], so too will the importance of structural health monitoring (SHM) in ensuring wind power reliability [4]. On land, wind turbines are typically supported by gravity-based, reinforced-concrete foundations. The ongoing health of these foundations depends on decisions and errors made during design; shrinkage and thermal gradients during casting; and subsistence and chemical attack during service [5,6]. As with any structure, the stages of damage identification in foundation SHM include: (1) detection; (2) location in time and space; and (3) an assessment of the severity of damage [7]. Large-scale sensor networks to solve the detection and location problems in civil structures are becoming increasingly common. Like bridges and towers [8,9], wind turbine foundations can be instrumented with sensors to assess and ensure ongoing structural health and safe operation [10,11]. Once sensor data are acquired, various techniques can be used for signal or statistical damage identification: Hilbert-Huang transforms [12], independent component analysis [13] and Bayesian approaches [14] being the most common. To quantify damage severity, however, most model-based approaches in SHM require estimates or prior knowledge of the system’s pristine state before the damage has occurred. In the absence of this data, any initial estimates of damage severity are at risk of being vague, and this can impact the relaibility of structural health forecasts [15].

For reinforced-concrete structures, one of the most important initial conditions and quality measures is compressive strength. A concrete’s strength is not just a direct measure of its ability to support load, but also an indirect measure of its elasticity and durability [16,17]. As such, there is clear value in non-destructively evaluating wind turbine foundation strength, particularly as these structures are subjected to large, continuous and dynamic loading patterns. In this work, we demonstrate that measurements of internal foundation temperatures, taken during curing, can be used to estimate the development and distribution of concrete strength within a real wind turbine foundation. The ‘maturity methods’ used in this work are based on the principle that the temperature profiles measured in the foundation during curing are the direct result of heat generated by the chemical reactions responsible for concrete strength. Field assessments of distributed temperatures in foundation structures, including wind turbine foundations, feature in several previous case studies [18,19,20,21]. However, these studies used sparse networks of (five or fewer) thermocouples to assess residual thermal stresses and shrinkage, not strength development. Indeed, during foundation curing, it is now standard practice for operators to record internal concrete temperatures in four locations to ensure thermal stresses are minimal. To find examples of applied maturity methods, we must turn to civil megastructures, such as bridges [22] and dams [23,24]. In recent years, there have also been several demonstrations of laboratory and field applications of wireless sensor networks for the maturity monitoring of buildings, pavements and slabs [25,26,27,28]. However, the prime focus of many of these studies has been on the development of new sensor technologies, and as such these studies: (i) do not verify estimated strengths by testing samples taken from real structures; and (ii) use strength models developed for traditional concretes, which do not accurately describe concretes containing modern additives such as fly ash [29].

In the work outlined here, we address these shortcomings by augmenting operator foundation thermocouple measurements with a dense, high-resolution network of 11 additional wireless thermocouples. Temperature data are used with both historical and recently developed methods in fly ash concrete strength and maturity modelling to provide, for the first time, an estimate of the temporal and spatial dependence of the strength distribution within a wind turbine foundation. In a step that we believe is unique to this study, we also verify our strength estimates with core samples. Finally, we extend the study by using the data obtained to train an artificial neural network, which after training, can predict in-situ concrete strengths independently of the supplementary data from the wireless thermocouples (i.e., using only sparse measurements from the operator). These results could provide models and other SHM techniques with more accurate estimates of the initial states and mechanical properties of concrete foundations [30]. The study has other practical benefits too: better knowledge of foundation strength development could facilitate time- and cost- savings from design through to construction and maintenance [31]. Meanwhile, the rapid and automatic analysis provided by the neural network could help operators make better use of their existing measurements, without the added costs of managing larger sensor networks and their associated data burden [32].

The methods which we outline here are not the only non-destructive testing techniques for monitoring in-situ concrete strength. Other examples include monitoring the transmission or reflection of ultrasonic waves [33], the harmonic response of vibrations in embedded piezoelectric transducers [34,35] or electromagnetic impedance [36]. The advantages of using the thermal approach outlined in this paper are the low sensor cost, the fact that thermal effects are more likely to be understood by practising civil engineers, and that temperature measurements can be more conveniently interpolated beyond sensor locations.

This paper begins with a description of the theory behind the maturity and strength modelling of fly ash concrete in Section 2. The field installation and analysis procedures are then outlined in Section 3, before the results and discussion are presented in Section 4.

## 2. Theory

**Figure 1:**
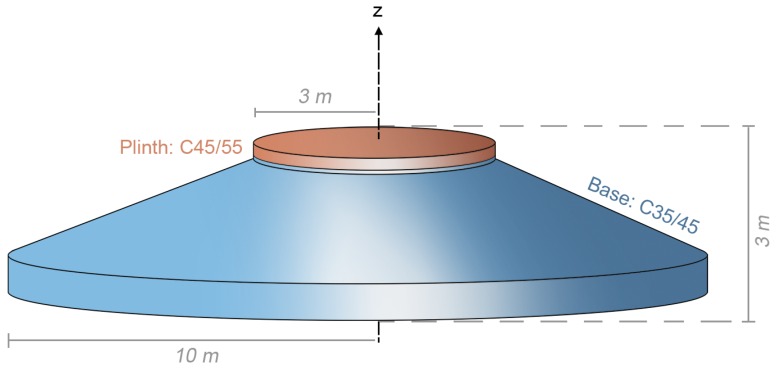
Illustration of the reinforced-concrete wind turbine foundation studied in this work.

The gravity-based wind turbine foundation studied in this work was of a circular, reinforced concrete design, as illustrated in Figure 1. In this design, a 20 m diameter base supports a 6 m diameter plinth, which is coupled to the tower using prestressed bolts. The foundation was cast on site, mostly from C35/45 concrete (i.e., concrete designed to have a 28-day cube strength of 35–45 MPa). The mix design for this concrete is provided in Table 1. To ensure adequate performance at the tower-foundation interface, the top 300 mm of the plinth was cast using a C45/55 concrete. This concrete had a similar mix design, but the higher strength was achieved by slightly reducing the ratio of water to cement.

### 2.1. Hydration Reactions

**Figure 2:**
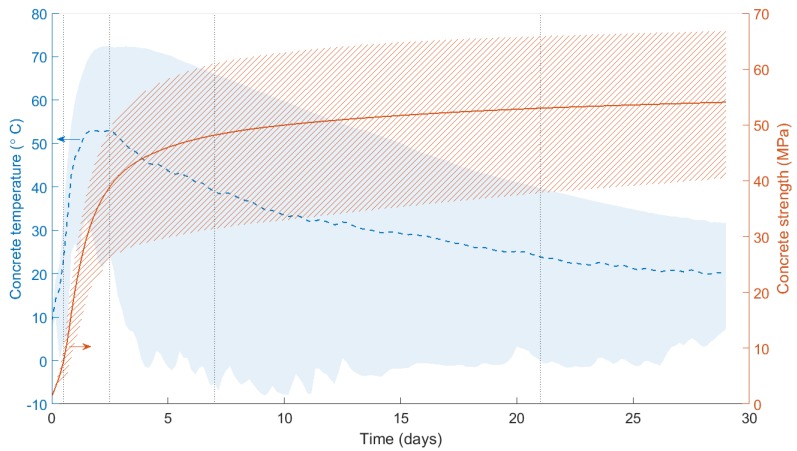
Mean concrete temperature and strength for the 29 days after foundation casting. Plotted data and boundaries represent the average, maximum and minimum temperatures monitored in this work.

Shortly after freshly-mixed, wet concrete is placed, the alite and belite present within the ordinary Portland cement (OPC) undergo exothermic hydration reactions with water. This produces calcium silicate hydrate (CSH), a gel which binds the suspended aggregates and is responsible for the majority of concrete’s strength. Figure 2 illustrates a very typical temperature signature for a concrete specimen as it cures and matures: heat is released from the hydration reactions, leading to a peak in concrete temperature and a rapid rise in concrete strength. As reactants and water are used up or lost to evaporation, the rate of hydration slows, leading to a decay in temperature and a reduction in the strength gain rate.

In this work, 30% of the OPC within the concrete was replaced with pulverised fuel ash (PFA), also known as ‘fly ash’. As fly ash is a pozzolan, it produces CSH by undergoing a delayed and endothermic reaction with calcium hydroxide (or free lime, a product of the main hydration reactions [37]). As a result, replacing a portion of the OPC with fly ash will: (i) slightly reduce initial strength gains; (ii) limit maximum temperatures within the curing concrete; and (iii) enhance the delayed strength gains after 28 days. In this work, a set-retarding admixture was also added to further reduce the heat generated during curing, as high temperatures can cause the concrete to undergo shrinkage, thermal stresses and cracking.

### 2.2. Maturity Methods

It is clear that temperature plays a key role in concrete strength, as heat is both a product and accelerator of the reactions responsible for producing CSH. Indeed, so-called ‘maturity methods’ assume that the level of curing (and so the strength) of a concrete specimen can be derived solely from its age and temperature history. This allows the strength development of real concrete structures to be estimated from a combination of laboratory cube strength tests and temperature measurements in the field.

Broadly speaking, there are three steps in applying maturity methods for any given concrete mix and structure. Firstly, the relationship between concrete cube strength and age is established for controlled curing temperatures (in this work, 22 ${}^{\circ }$C) in lab conditions (see Section 2.2.4). In the second step, on-site temperature measurements are used to estimate the ‘equivalent age’ or maturity of the concrete cured in the field (see Section 2.2.1). Finally, the cube strength calibration is used with the equivalent age measurements to estimate in-situ strength. The cube strength tests are required because, in practice, a concrete’s strength development cannot be predicted from its mix design alone. The reasons for this include: (i) physical and chemical variations in the sourced materials that comprise the concrete mix (the cement, aggregates, water and additives); (ii) human error; and (iii) deliberate changes to the mix design that are made to achieve the desired workability and setting time in response to changes in weather or pouring delays [38].

#### 2.2.1. Calculating the Equivalent Age

A concrete structure’s maturity after curing for some time, $t^{\prime }$, can be expressed as an equivalent age, $t_{e}$. The equivalent age is the amount of time that a concrete cube would have to cure in lab conditions to reach the strength that the structure has attained in field conditions. This is defined mathematically as:

(1) $$t_{e}=\sum_{t=0}^{t^{\prime }}\alpha \left(T\right)\Delta t$$

where Δt is the time interval between temperature measurements, and the *age-conversion factor*, α(T), is a temperature-dependent scalar that is calculated using either Nurse-Saul or Arrhenius maturity methods.

#### 2.2.2. Nurse-Saul Method

The Nurse-Saul maturity method defines the age-conversion factor as:

(2) $$\alpha_{ns}=\left\{\begin{array}{cc} \frac{T\left(t\right)-T_{0}}{T_{R}-T_{0}} & :\;T(t)\geq 0\\ \;0 & :\;T(t)<0 \end{array}\right.$$

where T(t) is the measured concrete temperature at time *t*, while $T_{0}=$ 0 ${}^{\circ }$C and $T_{R}=$ 22 ${}^{\circ }$C are datum and reference temperatures, respectively. This equation implicitly assumes that the maturity gained is linearly proportional to the curing temperature, and that concrete stops maturing at or below 0 ${}^{\circ }$C. The Nurse-Saul equation was originally formulated to describe experimental observations [39]. It is valued in industry for its simplicity [40], and provides accurate predictions of pre 28-day concrete strengths, provided temperatures remain within a range of 20–50 ${}^{\circ }$C [41].

#### 2.2.3. Arrhenius Method

The Arrhenius maturity method provides more accurate estimates of the age-conversion factor for temperatures outside of a 20–50 ${}^{\circ }$C range [41]:

(3) $$\alpha_{ar}=exp\left[\frac{-E_{0}}{R}\left(\frac{1}{273+T(t)}-\frac{1}{273+T_{R}}\right)\right]$$

Here, $E_{0}$ [J/mol] is the activation energy of the hydration reactions and R=8.314[J/(mol·K)] is the universal gas constant. The Arrhenius method is often favoured in scientific literature as it is accurate over a wide range of curing temperatures and has a grounding in reaction kinetics [42]. The method is, however, sensitive to measurement errors at high temperatures [43] and requires knowledge of the activation energy, $E_{0}$. Typical values for $E_{0}$ are in the range 35–45 kJ/mol, but the actual value is dependent on the type of cement, and the type and quantity of other additives such as fly ash. In practice, $E_{0}$ can be calculated for a given concrete mix by analysing the early-age strength development at a variety of curing temperatures [39], or it can be estimated from average curing temperatures [44]. In this work, we opt to use an alternative and updated method for estimating fly ash concrete activation energies, as forward by Kim et al. [45], and outlined in Section 2.2.4.

#### 2.2.4. Strength Dependence

A calibration curve between concrete cube strength, *S*, and curing time, *t*, can allow field-measurements of equivalent age to be converted into estimates of compressive strength. The most well-known method for fitting the relationship between data on *S* and *t* is the hyperbolic method, featured in ASTM C1074 and put forward by Carino in 1984 [46]:

(4) $$S\left(t\right)=S_{u}\frac{k_{th}\left(t-t_{0}\right)}{1+k_{th}\left(t-t_{0}\right)}.$$

Here, the fitting parameters are $S_{u}$, the limiting strength of the concrete (or its compressive strength at a theoretical ‘infinite age’) and a rate constant $k_{th}$ [days${}^{-1}$]. The constant $t_{0}$ is the time at which strength begins to develop (usually assumed to be $t_{0}=0$ days). Parameters can be estimated from a non-linear fit of Equation (4), or as is often the case in practice, they can be calculated from a linear fit of $\frac{1}{S}$ vs. $\frac{1}{t}$, as:

(5) $$\frac{1}{S(t)}=\frac{1}{S_{u}k_{th}}\frac{1}{t}+\frac{1}{S_{u}}.$$

Carino’s equation accurately models the strength dependence of traditional OPC based concretes before 28 days. It is, however, less accurate for late stage strengths, particularly for concretes which contain fly ash, as these introduce added and delayed strength gains from pozzolanic activity [47]. As such, Kim et al. proposed an improved method [45] which they have demonstrated is valid for modelling the late stage strength dependence of fly ash concrete [29]:

(6) $$S=S_{u}\left(1-\frac{1}{\sqrt{1+A\left[exp\left(-\frac{E_{0}}{RT(t)}e^{-\alpha t}\right)+exp\left(-\frac{E_{0}}{RT(t)}e^{-\alpha t_{0}}\right)\right]\left(t-t_{0}\right)}}\right).$$

Here, $E_{0}$ and T(t) are the activation energy and curing temperature, respectively (in Kelvin [K]). Meanwhile, *A* and α are constants which describe, respectively, a pre-exponential factor and a rate constant for how rapidly the activation energy diminishes. These factors can be intuitively understood as characterising the rate at which the reactive components within the concrete are used up. The form of Equation (6) is more complicated than Carino’s equation, but in addition to improved accuracy, its fit also provides a useful estimate of the activation energy. This estimate for $E_{0}$ can be substituted into Equation (3) to calculate the Arrhenius equivalent age of the concrete in the foundation.

## 3. Field Installation and Analysis

In this section, we outline the methods behind the field installation of the wireless thermocouples, the concrete coring and cube tests, and the analysis techniques used to obtain distributed and time-dependent estimates of concrete foundation strength.

### 3.1. Field Work

#### 3.1.1. Sensor Installation

**Figure 3:**
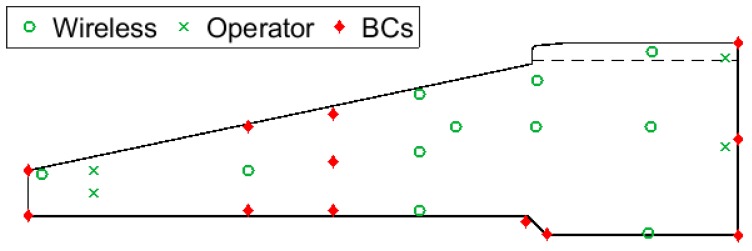
Axisymmetric representation of foundation illustrated in Figure 1, with thermocouples and boundary conditions (BCs, described in Section 3.2.3) highlighted.

After the steel reinforcement cage of the foundation was constructed, 11 wireless thermocouple probes with radio frequency identification (RFID) tags were installed in the locations highlighted in Figure 3. RFID tags were wired to the probes and placed 50–150 mm from the top surface of the concrete face. This ensured that the wireless signal was able to propagate to a receiver device, while maintaining adequate concrete cover over the reinforcement. Figure 3 also shows the four wired thermocouples installed as part of the wind turbine operator’s standard procedures (an additional thermocouple, not shown, monitors ambient temperatures). The temperature measurements of operator thermocouples are not routinely used for maturity monitoring, but are used to ensure that foundation temperatures do not exceed design limits, as this can cause large residual thermal stresses and cracking.

All thermocouples were installed along the same sector of the foundation, in the prevailing wind direction. The location of the wireless thermocouples was chosen to complement the readings from the operator’s probes and provide a more complete picture of the thermal gradients within the foundation. Between them, the thermocouples capture temperatures from the hottest points within the structure (at the centre of the pour around the plinth), to those closest to ambient temperatures (at the corner of the foot of the base). After the concrete was cast, all thermocouples logged hourly temperature measurements. These were stored on onboard memory and then downloaded after 14 days. Operator thermocouple measurements ceased after this period, but wireless thermocouples monitored for a further 14 days (29 days in total). The temperature resolutions of the wireless and operator probes were 0.1 ${}^{\circ }$C and 1 ${}^{\circ }$C, respectively.

#### 3.1.2. Concrete Pour and Coring

Concrete was poured around the reinforcement cage in a single day, from the outer edge of the foot of the base (i.e., the left hand side of Figure 3) up towards the plinth. A vibrating poker was used to consolidate the concrete and remove trapped air. The majority of the foundation was poured from C35/45 concrete, while the top 300 mm of the plinth was poured from a stronger C45/55 concrete mix. The interface between the two concretes is represented by the dashed line in Figure 3.

The foundation was backfilled 10 days after pouring. After 17 days of curing, three cylindrical core samples were taken from the very centre of the concrete base. These were compression tested as part of the operator’s regular inspection activities, and provided an opportunity to confirm the accuracy of our in-situ strength estimates.

#### 3.1.3. Cube Compression Tests

At the same time as foundation casting, approximately 100 cubes of side of 150 mm^2^ were cast from the same concrete mixes used during the pour (80 cubes from the C35/45 mix and 20 cubes from the C45/55 mix). After 24–72 h of dry curing at a mean temperature of 20 ${}^{\circ }$C, the cubes were placed into a water bath at 22 ${}^{\circ }$C to ensure adequate moisture for hydration (in accordance with standard BS EN 12390-2). At all times, cubes were protected from extreme temperatures to prevent errors in strength estimates [39]. Compressive tests on cubes were conducted after 1, 6, 17, 28 and 70 days, with sample sizes ranging from 2 to 42 cubes for each test.

### 3.2. Analysis

**Figure 4:**
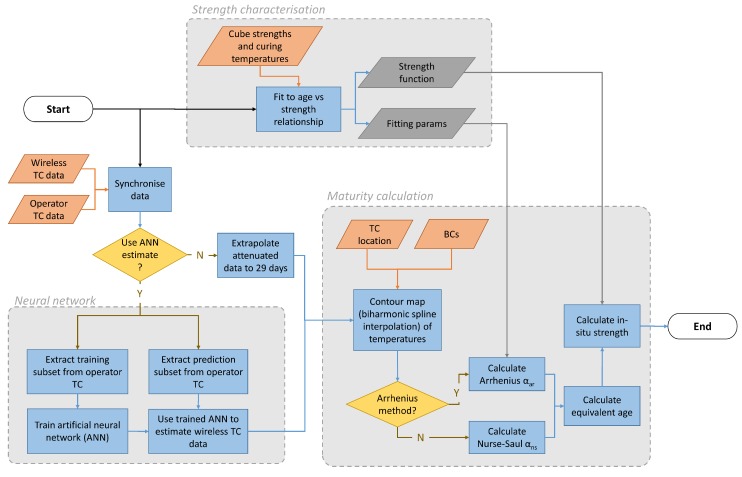
Flowchart summarising the analyses used to calculate in-situ concrete strength from thermocouple (TC) data.

The flowchart shown in Figure 4, summarises the analyses performed in this work. The first task is to establish the relationship between concrete cube strength and age for controlled curing conditions (Section 3.2.1). Distributed foundation temperatures are then extrapolated (Section 3.2.2) and interpolated (Section 3.2.3). Interpolation is produced using either a full dataset of temperature measurements, or a partial dataset of operator temperature measurements, extended using a trained artificial neural network (Section 3.2.5). Finally, equivalent age and in-situ strength calculations are performed (Section 3.2.4).

#### 3.2.1. Strength Dependence

For both the C35/45 and C45/55 concretes, the relationship between the equivalent age of the concrete cubes (assuming a reference temperature of $T_{R}=$ 22 ${}^{\circ }$C) and the strength results were fitted to the hyperbolic Equation (4) from Carino, and Equation (6) from Kim et al. Constant values of $t_{0}=0$ days and $A=10^{7}$ were used, as recommended in [29], while $E_{0}$, α, $S_{u}$ and $k_{th}$ were found by minimising the least squares error of non-linear fits. The fits were weighted using the inverse of the strength variance of each data point.

#### 3.2.2. Temperature Extrapolation

**Figure 5:**
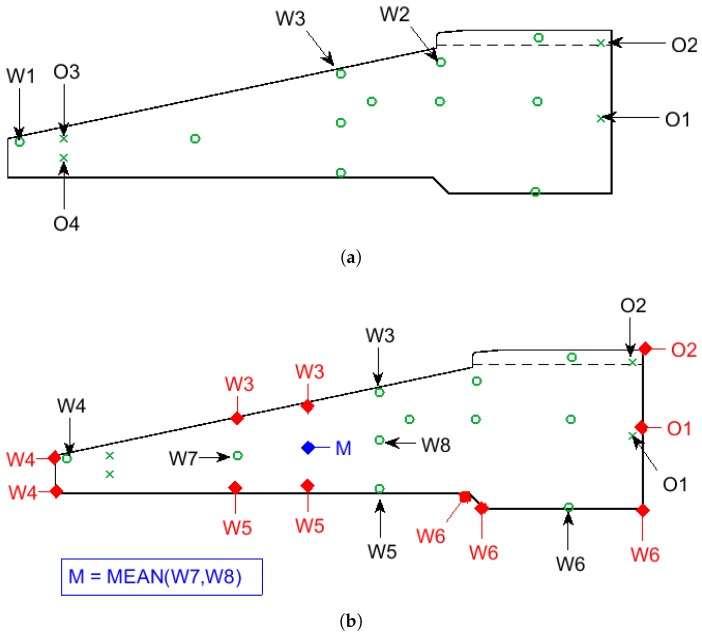
Thermocouple notation and boundary conditions and constraints used in this study. Note: ambient thermocouple O5 is not shown in these diagrams: (**a**) notation of thermocouples listed in Table 2 and Figure 6; and (**b**) boundary conditions applied to interpolation.

After the field trials had been completed, half of the thermocouples had recorded data for 29 days, while those listed in Table 2 had recorded data for 14 days. The notation used in this table is described in Figure 5a. Two wireless thermocouples (W1 and W2) failed after 14 days, while the operator’s thermocouples (O1–O4 and ambient) only recorded for 14 days in line with their standard procedures. To extend our analysis up to a 29 day period, the thermal decay data from the attenuated thermocouples were extrapolated. To achieve this, data between day 6 and day 14 were used for fitting. The thermocouples in the centre of the base (O1 and O2) showed an exponential decay. This is an expected result of Newton’s law of cooling, as thermal conduction with the surrounding concrete is the dominant heat exchange mechanism. The fitting exponents of these thermocouples reveal that O1, which is surrounded by a mass of hot concrete, shows a weaker thermal decay than O2, which is nearer the surface.

**Figure 6:**
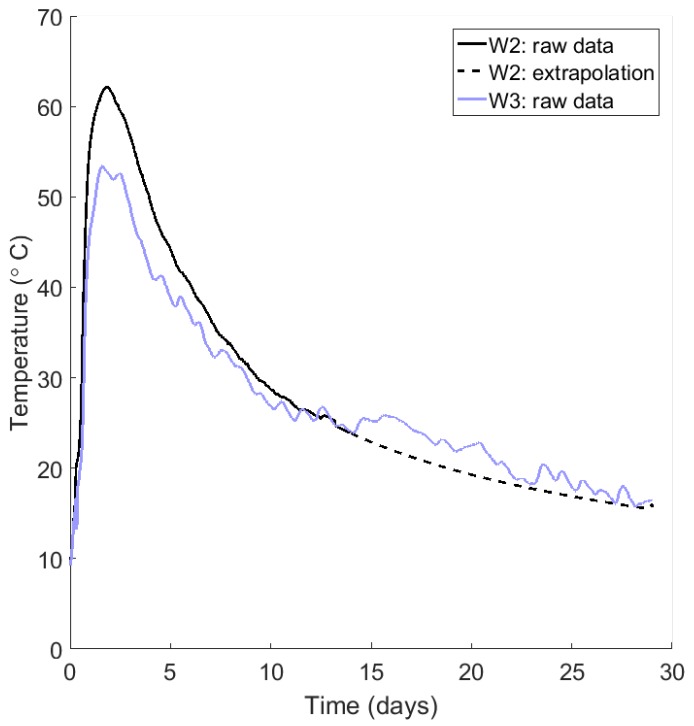
Example of the extrapolation of thermocouple W2, compared with nearby W3.

The remaining thermocouples are exposed to a superposition of heat loss mechanisms, including convection and radiation. As a result, a simple exponential decay does not apply, and a power-law decay provides a better fit. Although the extrapolations shown in Table 2 are nonlinear, the goodness of fit ($R^{2}$) values provided are valid as exponents were found from linear fits on logarithmic axes. Note also that extrapolated values were not allowed to fall below ambient temperatures (found from local weather data). Figure 6 shows an example of the extrapolated decay of thermocouple W2, compared to its nearest neighbour W3, which is in similar thermal conditions. Theoretically, W2 should not be cooler than W3 as it is nearer the centre of the base. However, as with most of the decays predicted, extrapolated temperatures are likely underestimated. This is a conservative approach as it leads to a lower strength predictions.

#### 3.2.3. Contour Mapping

Temperature data from the thermocouples were extended to map over the entire axisymmetric representation of the foundation through biharmonic spline interpolation. This produced contour maps of distributed foundation temperatures for each time point, i.e., values of T(X,Y,t), where $X(=x_{1},x_{2},\ldots ,x_{100})$ and $Y(=y_{1},y_{2},\ldots ,y_{100})$ were spatial coordinates within the bounds of the foundation map. These contour maps were used as the basis for subsequent equivalent age calculations.

**Figure 7:**
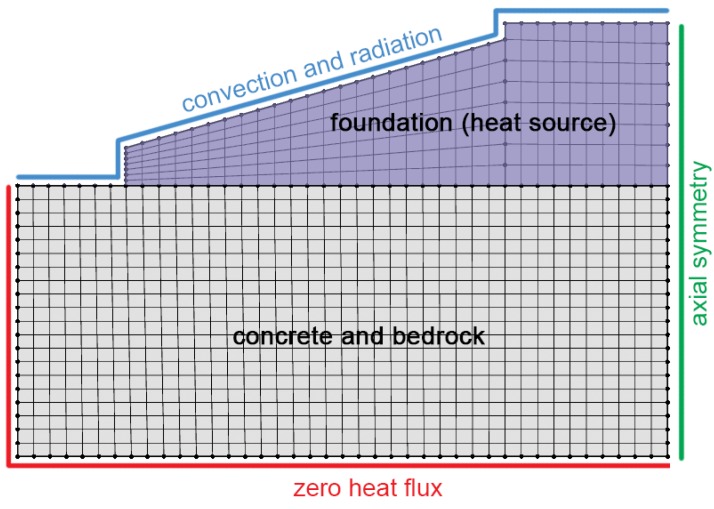
Thermal FEM mesh and boundary conditions.

**Figure 8:**
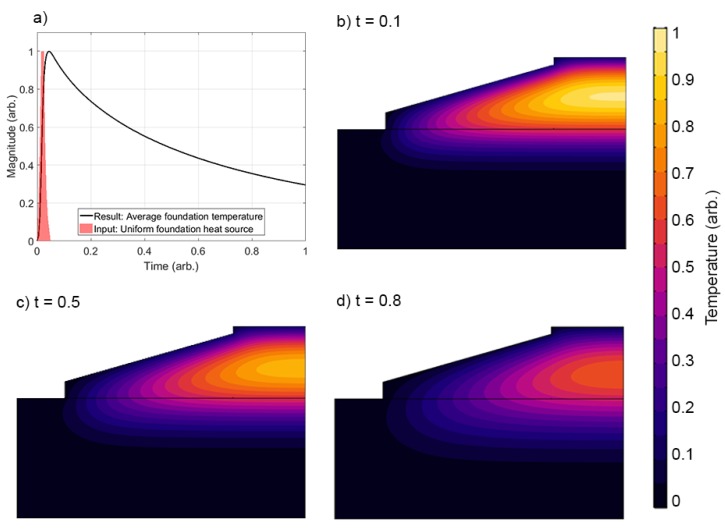
Thermal FEM results showing: (**a**) average foundation temperature results/input heat source; and (**b**–**d**) temperature profiles at various times.

There is no guarantee that an interpolation performed using the data from the sensor locations, shown in Figure 5a, will be sensible at the boundaries of the concrete foundation. Achieving this requires that the temperatures at the boundaries are propertly constrained. In this study, we achieve this by mapping temperature measurements from existing thermocouples to other locations in the foundation, as shown in Figure 5b. This mapping exercise was informed by a simple thermal finite element model (FEM) of the foundation system. The mesh and boundary conditions of the FEM are shown in Figure 7, while the parameters of the FEM are provided in Table 3. Convection and radiation were allowed to act over the exposed surfaces of the foundation and ground, while a zero heat flux boundary condition was applied to the volume underneath the foundation. The foundation itself acted as a uniform volumetric heat source [W/m^3^]. As shown by the shaded area in Figure 8a, heating was ramped exponentially to a maximum value of 10 kW/m^3^, at time $t_{arb}=0.01$, then held at a maximum value until $t_{arb}=0.011$, before decaying exponentially to zero by time $t_{arb}=0.05$.

The FEM has several simplifying assumptions. Firstly, note that the values provided in Table 3 are sensible estimates that are not based on properties measured in this field trial. Secondly, there is the assumption that heat generation within the foundation is uniform. In reality, the heat generated by the hydration reactions and the time that this power is sustained for will depend on local temperatures. Both of these assumptions can be made in this instance as: (i) the impact of non-uniform heat generation is averaged out by the large thermal inertia of the foundation; and (ii) the aim of using the FEM was not to gather numerical solutions (as our field measurements would achieve this) but to understand the temporal and spatial dependence of the temperature profile so that we could perform the boundary condition mapping shown in Figure 5b. The results are therefore not claimed to be numerically exact, and so are provided in arbitrary units.

The results of the FEM are shown in Figure 8. The average foundation temperatures have a similar time profile to that measured by thermocouples in the real base, shown in Figure 2. The distributions of temperature, meanwhile, agree with similar studies conducted for embedded ring foundations [18]. Temperatures rapidly rise within the centre of the foundation, before sinking towards the bottom of the base. The reason for this is that the concrete blanking and rock below the foundation act as thermal trap, which release heat back to the foundation at later times.

**Figure 9:**
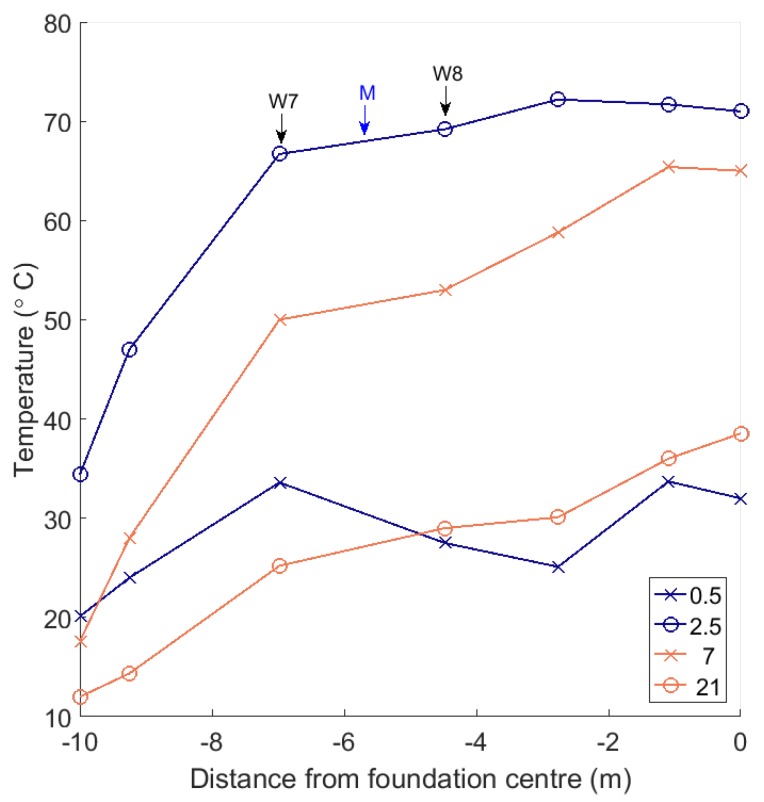
Central temperatures measured in the radial direction. Figure 5 shows the locations of labelled thermocouples W7 and W8, and the constraint M.

The results of the FEM allowed us to apply boundary conditions (BCs) to constrain the interpolation of the results measured by the wireless thermocouples in the field. Figure 5b shows the locations where boundary conditions (BCs) were applied. At BC locations, temperatures were mapped across from the data of thermocouples in similar thermal conditions (as shown, this is typically a near neighbour). With the exception of the point labelled ‘M’ in Figure 5b, the interpolation algorithm needed only be restricted at the edges of the foundation. The restriction at the point labelled ‘M” is to prevent the interpolation from estimating low temperatures in the region between W7 and W8. Taking the mean value of W7 and W8 at this point is equivalent to linear interpolation. This action was supported by the FEM results, and by the trends in our thermocouple measurements, displayed in Figure 9.

#### 3.2.4. Equivalent Age and Strength Calculations

After foundation temperatures had been established, Nurse-Saul and Arrhenius age-conversion factors were calculated using Equations (2) and (3), respectively. Equivalent ages were calculated using Equation (1) and a time step of Δt= 1 h. Finally, the strength fitting, outlined Section 3.2.1, allowed equivalent ages to be converted to estimates of in-situ concrete strength.

#### 3.2.5. Artificial Neural Network

As an extension to the study, we investigated whether operator thermocouple data could alone be used to estimate distributed foundation temperatures, i.e., *without* the supplementary information from wireless thermocouples. This could in principle allow the operator to estimate distributed foundation strengths over other foundations without altering their standard operating procedures.

**Figure 10:**
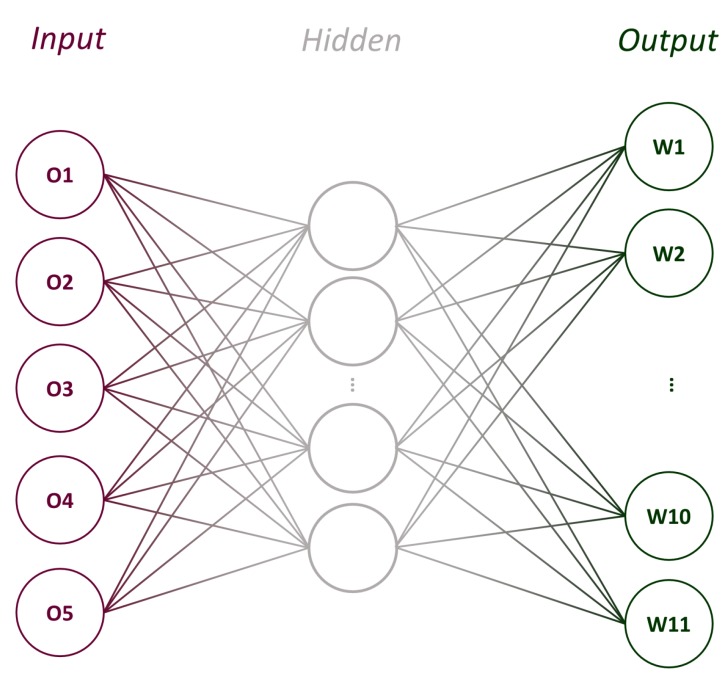
Illustration of ANN with a hidden layer (of 10 nodes, not all shown). Five inputs (operator thermocouples O1–O5) are used to predict 11 outputs (wireless thermocouples W1–W11).

**Figure 11:**
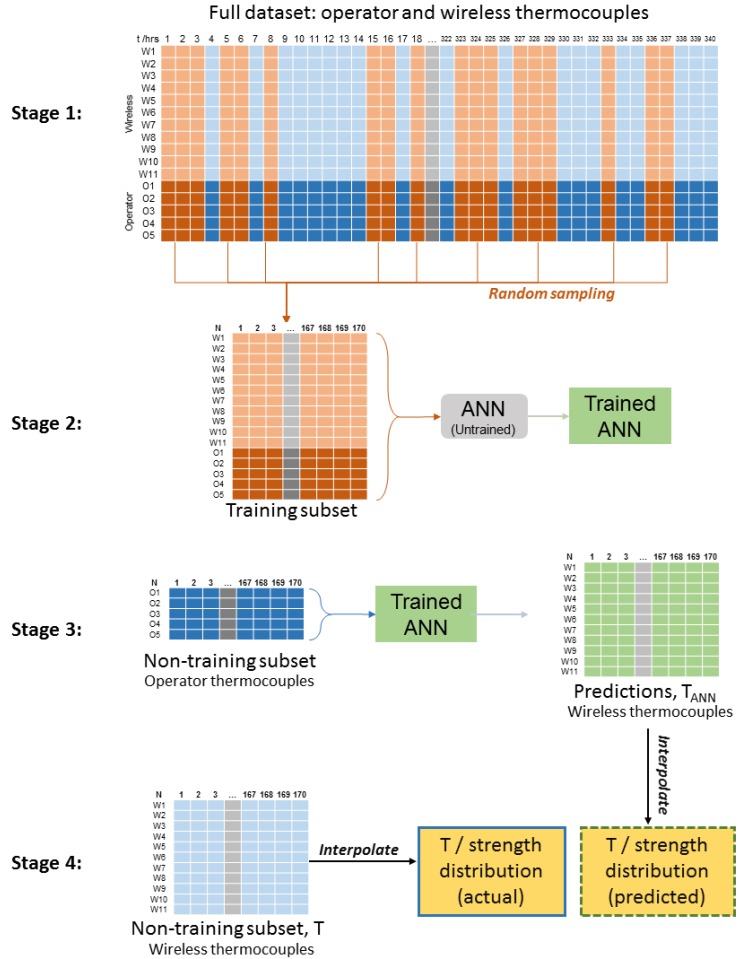
Graphical summary of the four stages used to train an ANN and then use it to predict distributed temperatures in the foundation.

To achieve this, we used an artificial neural network (ANN)—an effective tool for minimising errors in complicated non-linear fits. The ANN used Levenberg-Marquardt training and one hidden layer made up of 10 neurons, as illustrated in Figure 10. In this case, the data was not extrapolated as outlined in Section 3.2.2, and only the available 14 days (340 h = 340 samples) of data were used. The four-staged method used to train, verify and then use the ANN in this work is summarised in Figure 11. Of the 340 samples, the network was trained using a random subset of 50% of the samples (see Stages 1 and 2 in Figure 11). More detail on the training of ANNs can be found in [50], but briefly, the inputs (data points from O1–O5) are randomly weighted and summed at each node in the hidden layer, before being passed through a non-linear ‘activation function’. This happens again between the hidden layer and the output. The 11 outputs are compared against expected values (wireless thermocouple data W1–W11), so that the error can be gradually minimised by re-tuning the weightings.

After 170 data points of training data, the weightings are fixed at their optimum values and the final performance of the network is validated using the remaining 170 samples (Stage 3 in Figure 11). During validation, the recorded wireless thermocouple temperature values are denoted $T_{i}$ for i=1,2,…11, while the ANN’s predictions are denoted $T_{ANN,i}$. It follows that the ANN’s error (or over-prediction) is:

(7) $$\Delta T_{op,i}=T_{ANN,i}-T_{i},$$

This is the metric used to define the performance of the ANN. Once temperatures have been predicted, interpolation can be used to estimate the distribution of foundation temperatures (or strengths) from the ANN’s predicted values. This can be compared with the distribution that would have been obtained using actual data (Stage 4 in Figure 11). Note that using ANN-predicted values for strength calculations requires that the time elapsed between the randomly sampled validation data points is accounted for by changing the value of Δt in Equation (1) to match the new time differences between data points. Note also that the strengths calculated from ANN predictions, $S_{ANN,i}$ can be compared with the strengths predicted from real wireless thermocouple data, $S_{i}$, similar to Equation (7). This produces a second metric to define ANN performance: a set of strength errors, $\Delta S_{op,i}$.

## 4. Results and Discussion

### 4.1. Cube Result Strength Models

**Figure 12:**
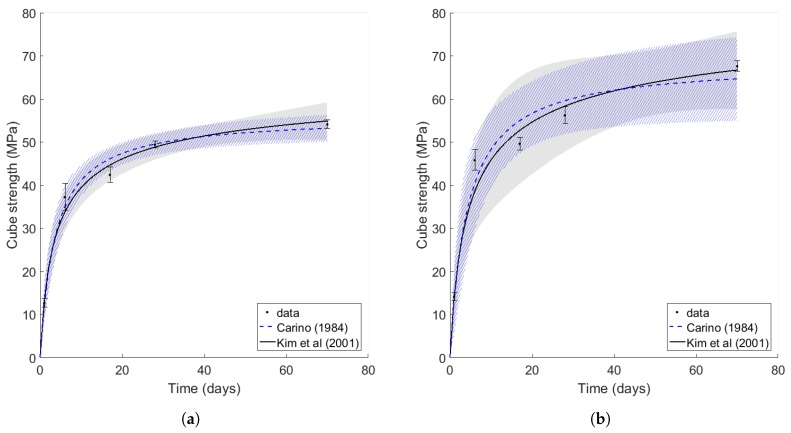
Cube strengths with fits based on equations from Carino (dashed line, hatched area) and Kim et al. (solid line, solid area): (**a**) C35/45 concrete mix and (**b**) C45/55 concrete mix. The shaded areas are the 95% confidence intervals for the fits.

Cube strength results for the C35/45 and C45/55 concrete mix are shown in Figure 12a,b, respectively. The fits using, respectively, the methods by Carino, and Kim et al. are shown along with the 95% confidence interval of each fit. The fitting parameters for both concrete mixes are provided in Table 4. It is clear that the fit has been successful for the weaker concrete mix, but that the fit for the stronger mix is made with a lower confidence. This may be because the smaller sample size used in the C45/55 study does not allow the mean values of the strength to be calculated with high certainty.

Nevertheless, the fit from Kim et al. outperforms that of Carino in its predictions of the strength gain rate of the fly ash concretes used. It successfully captures the fact that some early stage strength is sacrificed for late stage, pozzolanic strength, and it furthermore provides sensible estimates for the activation energy of each concrete mix. As such, the Kim et al. fit is used as a calibration curve for subsequent foundation strength predictions in this work.

### 4.2. General Trends in Temperature and Maturity

**Figure 13:**
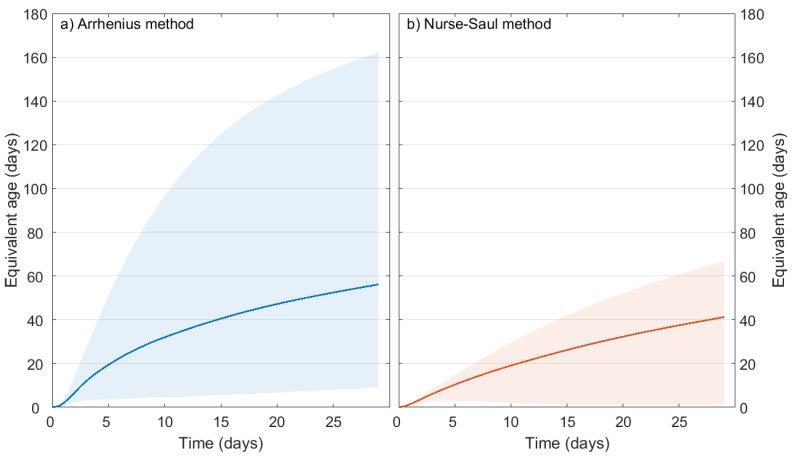
Average and (shaded) maximum and minimum foundation equivalent age values calculated using: (**a**) Arrhenius and (**b**) Nurse-Saul methods.

Figure 2 shows the mean, highest and lowest recorded concrete temperatures and calculated strengths in the foundation for the 29 days after casting. The temperature and strength signature is typical of bulk concrete body behaviour. In this case, peak temperatures occurred after 2.5 days, and the majority of the strength was gained within the first week. Figure 13, meanwhile, shows a comparison of the bulk equivalent age of the foundation calculated using: (a) Arrhenius and (b) Nurse-Saul methods. The shaded areas show the upper and lower bounds (i.e., the most and least mature sections of concrete in the foundation). It is clear from these results that the Arrhenius method more accurately captures the range of concrete maturities present in the foundation, from the higher maturities in the centre of the base, to the lowest maturities at the edges exposed to ambient conditions. This highlights the value of the Arrhenius method in a system where temperatures regularly vary from 10 ${}^{\circ }$C to 70 ${}^{\circ }$C. As such, the Arrhenius method is used for the remainder of the results presented in this paper.

### 4.3. Temperature Maps

**Figure 14:**
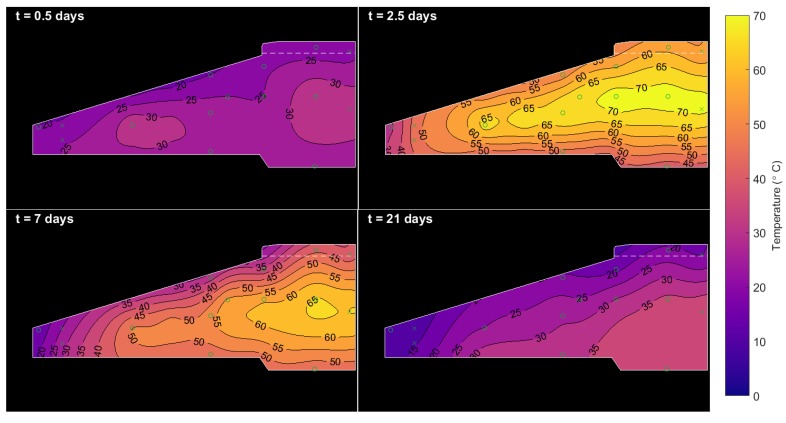
Contour maps of foundation temperatures during the first 22 days after casting.

Figure 14 shows snapshots of the temperature map in the foundation after 0.5, 2.5, 7 and 21 days (these times are shown by vertical dashed lines in Figure 2). As expected, temperatures build rapidly to a peak of 70 ${}^{\circ }$C within the centre of the pour over the first few days before ebbing away from the top surface of the foundation. As predicted by the FEM, heat loss is particularly pronounced over this top surface because it is exposed to air, while the bottom surface is insulated by a concrete blanking slab, and surrounding rock and soil. The high thermal inertia of the foundation allows it to retain temperatures as high as 35 ${}^{\circ }$C for at least 21 days. While it is outside of the scope of the current study, the temperatures collected in this work could be used to calculate residual thermal stresses in the concrete foundation (as in [18]), as these can be responsible for crack initiation that is exacerbated by loads during operation.

Figure 9 shows the radial dependence of the temperatures measured by centrally located thermocouples in the foundation. The trends on days 0.5, 2.5, 7 and 21 are shown. The most interesting behaviour is after 0.5 days of curing, as the outermost thermocouples register higher temperatures than those towards the centre of the foundation. This is likely a consequence of the fact that the foundation is poured from the outside inwards, so the concrete at the outermost region has more time to heat up. The slightly higher temperatures in the foot do seem to persist until later days, but overall, there is a negligible effect on strength once the pour is complete.

### 4.4. Equivalent Age and Strength Maps

**Figure 15:**
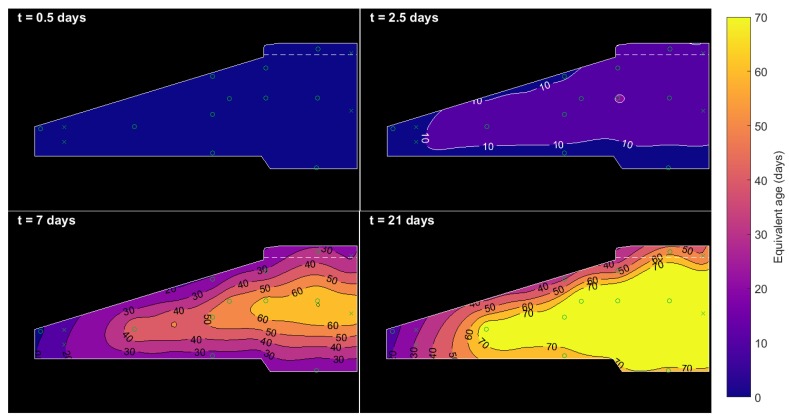
Progression of Arrhenius equivalent age. Contours are limited to a maximum of 70 days.

**Figure 16:**
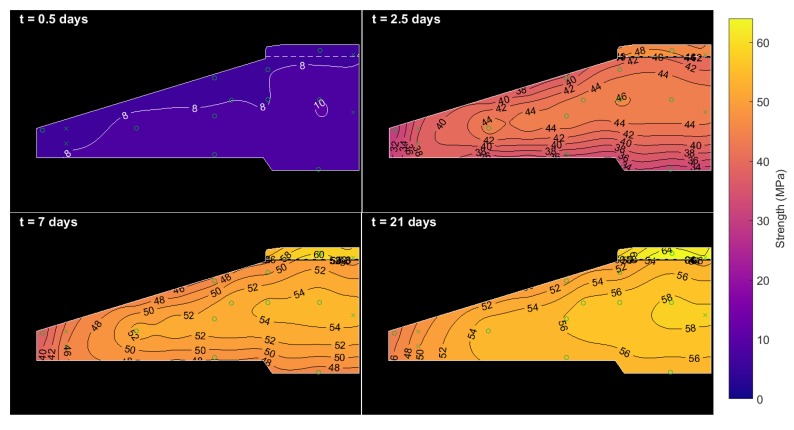
Strength progression in the foundation. Contours are limited to a maximum of 65 MPa.

Figure 15 and Figure 16 show a progression of contour maps for Arrhenius equivalent age and calculated concrete strength. The equivalent ages and strengths shown in the figures are not plotted beyond 70 days and 65 MPa, respectively, as these are close to the boundaries of the fit from Section 4.1. It is clear that, within a week of casting, the concrete at the centre of the pour rapidly matures at high temperature to an equivalent age of 60 days, and the majority of both types of concrete surpass their design strength (even when accounting for the reasonably large confidence interval of the C45/55 concrete mix fit, shown in Figure 12b). The very tip of the foot of the foundation matures more slowly, particularly as it is not insulated or warmed by any surrounding concrete (or backfill within the first 10 days). If desired, these in-situ concrete strengths could be used to calculate the apparent distributed Young’s modulus of the concrete for design purposes. This follows from American building code ACI 318, where concrete elasticity is stated as $E_{c}\propto \sqrt{S}$.

**Figure 17:**
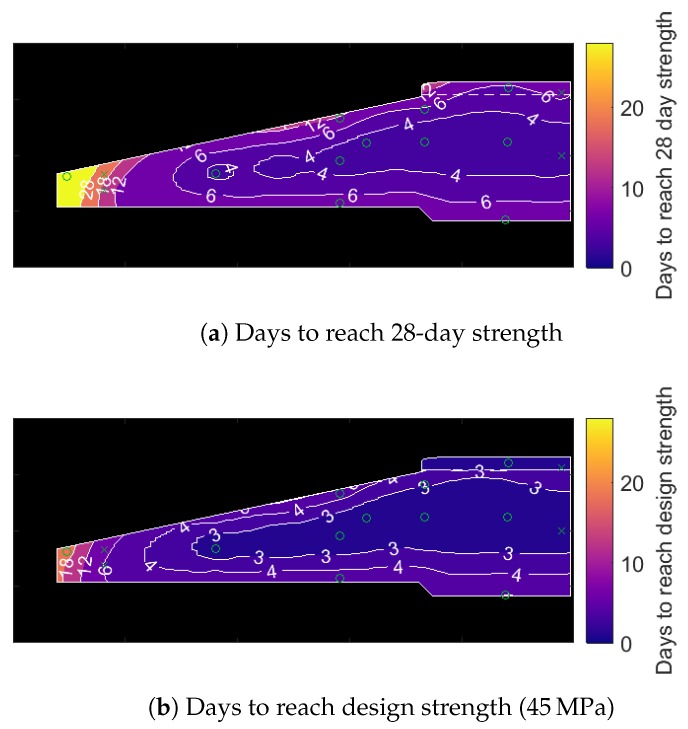
The number of days required for the concrete in the foundation to reach a given strength benchmark.

An operator may be more interested in knowing when the foundation reaches its 28-day strength or its design strength. These quantities are plotted in Figure 17a,b, respectively. The 28-day strengths are taken from Figure 12, while design strengths are assumed to be 45 MPa for the entire foundation. As shown, both measures of strength are achieved for all but the tip of the foot of the foundation within 12 days. The tip of the foot of the foundation is more exposed to ambient temperatures (in this case, around 10–15 ${}^{\circ }$C) and so takes 18–22 days to reach design strength, or beyond 30 days to reach ‘28-day strength’. This latter result is unlikely to be of concern because: (i) surpassing design strength is more important; and (ii) this volume of concrete is not subjected to large loads.

### 4.5. Core Samples

The compressive strengths of the three core samples taken from the mid-point of the foundation after 17 days were 62±2 MPa. The operator’s most centrally located thermocouple (labelled O1 in Figure 5a) recorded temperatures in the vicinity of the cored location. As such, measurements from this thermocouple were used to estimate strengths using the Arrhenius maturity method. The equivalent age after 17 days was 120 days, which was far beyond the point at which cube samples were taken. The strength model has a quantifiable confidence interval within the time range bounded by the data points, but extrapolation to later times is subject to unknown (and potentially large) errors. Nevertheless, the strength estimate corresponding to this equivalent age was 58 MPa, an estimate which is within two standard deviations of the experimental core strengths. A more conservative hypothesis is to state that we expected the concrete in this location to exceed its ‘70-day cube strength’—and this is something which has certainly been achieved.

In future work, better confirmation of core strengths and full quantification of the error could be achieved if: (i) cores were taken earlier; (ii) the cube strength study was extended over a longer period; or (iii) thermocouple measurements of O1 are taken for a longer period (rather than extrapolated as they were in Section 3.2.2). None of these actions are currently part of the operator’s standard procedures, but the latter two may be preferred as they are relatively simple to achieve. Extending cube tests up to 120 days would usually be of limited value due to the diminishing returns of concrete strength, but the pozzolanic strength gains of fly ash concretes may warrant the extra effort in future work. Other future work could include measuring environmental humidity and internal moisture in the concrete foundation, as demonstrated in laboratory work in [51]. Given water’s importance in concrete’s hydration reactions, accounting for its impact on in-situ strength development may improve our models of strength development.

### 4.6. Artificial Neural Network

**Figure 18:**
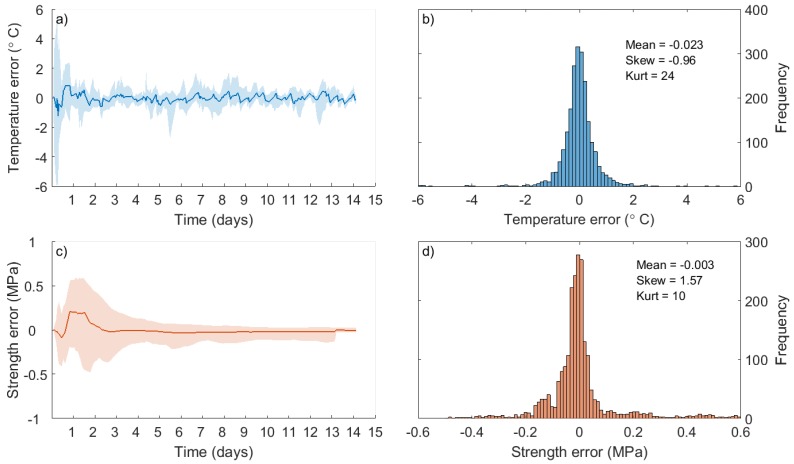
Temperature (**a**,**b**); and strength (**c**,**d**) over-prediction of the ANN.

As outlined in Section 3.2.5, a neural network was used to predict the temperature values of the 11 wireless sensors, using only the operator’s thermocouples as inputs. After training using half of the data points, the mean, maximum and minimum errors (over-prediction) of the ANN can be analysed for the remaining half of the data. These errors are shown for temperatures in Figure 18a and for strengths in Figure 18c. Meanwhile, histograms of errors for temperature and strength are shown in Figure 18b,d, respectively. While the results presented in Figure 18 are for a particular random sample, the outcome is very typical. Mean errors are typically low, but there is a slight over-prediction of temperature prior to day 2. This is likely a consequence of the fact that temperatures are rapidly rising and the behaviour of the system is changing: the issue could be solved by sampling more rapidly during the initial temperature increase of the foundation, as this would provide a larger training dataset in this region. Nevertheless, as the model stands, the resulting fractional errors in the strength calculation are consistently below 0.1%.

The method has clearly been successful for this foundation: contour plots of temperatures, equivalent ages and strengths using ANN predictions are indiscernible from Figure 14, Figure 15 and Figure 16. However, the advantages and limitations of the ANN should be compared in light of alternative methods, the most frequently used of which would be a full numerical FEM. In this context, the disadvantages of using an ANN are that it is a black box technique, which provides no insight into the underlying processes behind why or how heat is generated and lost. As such, an FEM can be tuned to suit new circumstances, but an ANN cannot without new training data. It is likely that this neural network’s weightings will not be valid for foundations with different geometries or those made with different mix designs. It may even fail to accurately predict strengths for dramatically different weather conditions. The method is, however, much more convenient for the operator to use than an FEM, as it does not require any specialist (or expensive) software, and can be performed rapidly, in real-time if required. It is also not subject to the human errors or subjective bias that can undermine FEM calculations. What this exercise has demonstrated is that a trained ANN could allow operators to extract more value from their existing thermocouple data: to estimate distributed temperatures (and strengths) in other foundations by using the trained ANN (Stage 3 of Figure 11) without the added cost of installing a dense thermocouple network in every foundation. To reiterate, however, using this approach in practice would may require that the ANN be extended and verified with other training datasets, and other additional inputs.

## 5. Conclusions

This paper outlines the field application of up-to-date concrete maturity methods to a fly ash concrete wind turbine foundation. The methods described were able to estimate time-dependent and distributed concrete strengths throughout the foundation, based on in-situ wireless thermocouple measurements and cube compression tests. These strength estimates were partly verified by core samples, taken from the foundation after 17 days. In addition, the collected data were used to train an artificial neural network, which was able to provide accurate predictions of distributed foundation strengths based on sparse temperature readings from five low-resolution thermocouples. The acquisition of further datasets may allow for better training of this neural network so that it is able to predict temperatures, and strengths, for a wider variety of turbine bases. Overall, the proposed techniques could assist site operators in making more cost-effective decisions about foundation design, construction, operation, monitoring and maintenance.

## Figures and Tables

**Table 1:** Mix design for the C35/45 set-retarded concrete used in the foundation.

Dry Components	kg/m^3^
Cement: CEM II-BV, 70/30 PFA blend	400
Coarse aggregate 20–50 mm	1006
Fine aggregate (sand)	800
Free water	180
Superplasticiser	2
Set-retarding admixture	0.7

**Table 2:** Thermocouples which required extrapolation. The methods, exponents, and goodness of fit are provided.

Type	Notation (see Figure 5a)	Extrapolation	A	B	R^2^
Wireless	W1	At^B^	50	−0.48	0.852
	W2	At^B^	143	−0.66	0.998
Operator	O1	Aexp(Bt)	86	−0.037	0.974
	O2	Aexp(Bt)	64	−0.054	0.964
	O3	At^B^	105	−0.70	0.998
	O4	At^B^	111	−0.67	0.995
	O5 (Ambient)	Temperature records from local weather data			

**Table 3:** Parameters used in the FEM.

Parameter	Value	Units	Notes
Ambient temperature	293	K	Used for radiation and convective heat loss
Convective heat transfer coefficient	10	W/m^2^K	Assumed for natural convection
Emissivity of concrete	0.85		Used to calcualte radiation heat loss
Max heat power generation in concrete	10	kW/m^3^	Assumed to be uniform. Based on 4 mW/g max rate of hydration [48]
Concrete and bedrock density	2400	kg/m^3^	As outlined in [48,49]
Concrete and bedrock thermal conductivity	1.8	W/mK	
Concrete and bedrock heat capacity	880	J/kgK	

**Table 4:** For each concrete, the parameters found from least-squares fitting to Carino and Kim et al. equations.

	Carino (1984)		Kim et al., 2001		
	Su (MPa)	k${}_{\mathrm{th}}$ (days${}^{-1}$)	Su (MPa)	α	E${}_{0}$ (kJ/mol)
**C35/45**	56	0.28	63	6 × 10${}^{-4}$	42.5
**C45/55**	69	0.24	67	8 × 10${}^{-4}$	43.0

## Abbreviations

The following abbreviations are used in this manuscript:

CSH	Calcium Silicate Hydrate
FEM	Finite Element Model
OPC	Ordinary Portland Cement
PFA	Pulverised Fly Ash
RFID	Radio Frequency Identification
SHM	Structural Health Monitoring
